# Adaptive time-varying detrended fluctuations analysis: a new method for characterizing time-varying scaling parameters in physiological time series

**DOI:** 10.1186/1471-2202-12-S1-P105

**Published:** 2011-07-18

**Authors:** Luc Berthouze, Simon F Farmer

**Affiliations:** 1 Centre for Computational Neuroscience and Robotics, University of Sussex, Falmer, BN1 9QH, UK; 2 Institute of Child Health, University College London, London, WC1N 1EH, UK; 3 Institute of Neurology, University College London, London, WC1N 3BG, UK

## 

Detrended fluctuations analysis (DFA) [1] is a technique commonly used to assess the presence of long-range temporal correlations (LRTCs) in physiological time series. The method is based on assessing the parameters of the linear regression in the loglog space of the residuals of the detrended signal over different box sizes; providing an estimate of the Hurst exponent. Convergence of the method is asymptotic only [2] and therefore its application requires lengthy time series assuming a stationary scaling exponent. Methods for dealing with nonstationarities due to, e.g., data manipulation (e.g., stitching), addition of random outliers or the presence of different standard deviations or correlations assume the superposition of independent processes and rely on a graphical interpretation of changes in the slope of the residuals at various box sizes [3]. However, most neurophysiological experiments involve a task or neurophysiological perturbations. These may disrupt the LRTCs in unexpected and interesting ways. It is therefore of importance to devise a robust method for tracking changes in the parameter that best characterizes LRTCs.

We derived analytical formulations for the bias and variance of the error committed when using the scaling exponent obtained by DFA on a given time-series to predict the scaling exponent of the same time-series but shifted by one sample (assuming the scaling exponent is stationary at a very short time scale). These results make it possible to define a Kalman filter for tracking fluctuations in scaling exponents over a longer time scale. Estimates for the measurement noise of the filter are obtained by pooling DFA estimates of the signal across a small number of time shifts. Robust estimates of the state vector are obtained by augmenting the filter with a smoothing procedure.

**Figure 1:**
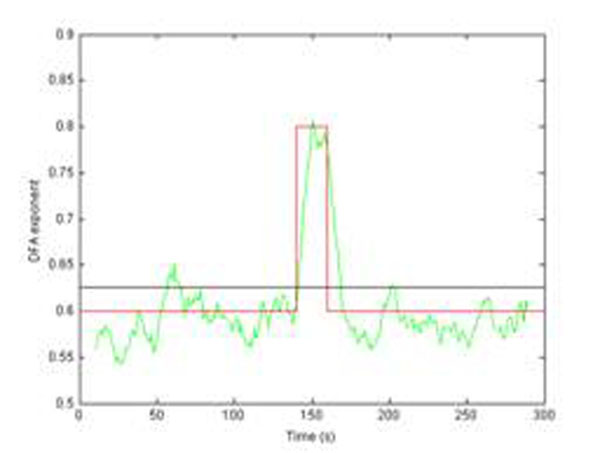
Comparison between standard DFA (black line, value obtained over whole record) and adaptive time-varying DFA (green line). The actual scaling exponent is shown in red.

Simulation results with surrogate time series demonstrate that this technique makes it possible to accurately recover changing scaling exponents even in the presence of rapid fluctuations (see Figure 1). It therefore provides a robust mechanism with which experimenters may be able to measure changes in the strength of LRTCs within their data. This may help move the debate concerning scale-free behavior of time series on from a simple demonstration of the existence of LRTCs to an appreciation of how their magnitude may vary systematically in response to experiments.
